# Hospital Work in East Anglia

**Published:** 1912-01-13

**Authors:** 


### HOSPITAL WORK IN EAST ANGLIA.

### A Retrospect of Seventy-Five Years.

THE East Suffolk and Ipswich Hospital, the premier institution of its kind in the county of Suffolk, is a continuation of the Ipswich Public Dispensary, which existed on the same site a century ago. The original building, opened in 1836, provided accommodation for forty patients, but considerable development has been effected in recent years, and the building now has a total of 141 beds. In the course of an interview the Secretary, Mr. Arthur Griffiths, gave a representative of THE HOSPITAL some interesting details regarding its development.

### THE HOSPITAL MAP.

In reply to a suggestion that the area of operations is a wide one, Mr. Griffiths replied, "Yes, it comprises the borough of Ipswich and 245 country parishes, with a total population of over 200,000. Of that number about one-third resides in the borough of Ipswich. The question of boundaries is usually a vexed one in hospital work, but the Board of Management in our case has solved the difficulty and avoided overlapping by means of a carefully prepared map showing the hospital's district. Although the constitution and statutes state that the hospital exists to afford medical and surgical aid to the suffering poor of every country and nation—a rather wide term, by the way—yet the management have a clearly defined area for operations, and are careful not to encroach on the territory of any other institution doing similar work."

"The hospital structurally has of recent years undergone considerable development, has it not?"

"Naturally, to keep pace with the progress of medical and surgical science, additions and improvements to our equipment are constantly necessary, besides which, with the growth of population, there is of course need for increased accommodation. Until three years ago the hospital was of the ordinary small county type, and had nothing to boast of in the way either of equipment or accommodation, but a masterful scheme of reconstruction and renovation was embarked upon by the management in 1907, culminating in 1910, and the institution now compares very favourably in every way with others in the country. The governors of the hospital have much to be thankful for. Some of the best-known men throughout the district have thrown themselves heart and soul into the work. Among them is Mr. P. E. Ripley, who has placed his great technical knowledge and engineering skill unreservedly at the disposal of the management, and has been the chief mover in the development of the hospital from the inception of the scheme, besides which he is a generous financial supporter of our work. The structural improvements and additions comprised in the scheme, upon which about £20,000 was expended, included a new administrative block with kitchens and offices, an operating theatre and anæsthetising room, a bacteriological department, isolation block, laundry, and mortuary. An electric lift, which greatly facilitates the movement of patients from one floor to another, a hot-water system, and improved heating apparatus were also installed."

### A PATRIOTIC BOARD.

"Since the completion of the work, a year since," continued Mr. Griffiths, "further additions have been made to the hospital. Mr. John D. Cobbold, a member of the Board of Management, and ever one of the institution's most liberal friends, has had erected as an adjunct to the children's ward—which, by the by, is a memorial to his father, the late Mr. John Patteson Cobbold, M.P.—open-air balconies on the most substantial and elaborate plan, affording permanent accommodation for sixteen further cots and increasing the number of beds in the children's ward to forty-five, a very fair ratio in a hospital with 141 beds. A porter's lodge, which might be described as a model building of its kind, has been given to us by the President, Dr. J. H. Bartlet, and occupation has just been taken by the head porter. Dr. Bartlet has also been a great benefactor to the institution. Not many years ago, in memory of his father, one of the original members of the honorary medical staff, he presented the fine, detached out-patients' department, near the entrance to the grounds, with its spacious waiting-hall and offices, and he has also recently endowed two beds."

"Now all this work has been accomplished, is there for the time being ample provision for your requirements?"

"On the contrary, despite the continual extension, every available bed is at this moment occupied, with the exception of a few reserved for accident cases, and, as you will see," said Mr. Griffiths, handing the waiting list for inspection, "there are some fifty patients there awaiting admission. This has been the case for three or four weeks past."

"There is already need for further enlargement?"

"That is so. The number of beds even now bears a very small ratio to the large population the institution is intended to serve."

"How could you further increase the accommodation?"

### THE BALCONY SCHEME.

"Well, the matter has not yet been considered, but the success of the open-air balcony scheme adopted for the children's ward, coupled with the magnificent geographical situation of the building, certainly leads one to suppose that the additional accommodation might be afforded to the main wards by means of balconies as it has been in the former case. These balconies might possibly run the whole length of the front of the main building, and all the rooms with a southern aspect could then be utilised for the patients. But that is a matter for wiser counsels than mine, and my first care is to provide the money for current expenses, which, with these legislative difficulties hanging like the sword of Damocles over my head, is sufficient worry for the present. The Board is now faced with a new difficulty, in that owing to the increased staff required through the extension, the accommodation in the nurses' home is insufficient."

"Have you anything to say about the governing body?"

"The affairs of the hospital are in the hands of a representative Board of Management, comprising representatives of workmen's organisations and Friendly Societies, as well as the usual subscribers and donors of large amounts. Unlike many hospitals, we do not exclude the medical staff from our counsels, and the acting Board consists of twelve elected members and three members of the honorary medical staff. The accounts are now presented on the Uniform System, with all the statistical tables in extenso. This method not only brings the institution into line with other large hospitals, but assists considerably in making comparisons with others, and forms a valuable check on the expenses of each department. A cursory glance at the accounts reveals the fact that the ordinary income for the past year was a record—£8,245 11s., or £92 more than the expenses, which speaks well for the support afforded to the hospital and the careful management."

